# Delayed-Onset PTSD and Coping Strategies of Chinese College Students During the COVID-19 Pandemic

**DOI:** 10.3389/fsoc.2021.734738

**Published:** 2021-10-27

**Authors:** Zhenxin Liao, Xueyan Zhang, Yingwen Wang, Tingwei Wang, Xinyu Li, Mingyi Zhao, Quan Zhuang

**Affiliations:** ^1^ Transplantation Center, The 3rd Xiangya Hospital, Central South University, Changsha, China; ^2^ Xiangya School of Public Health, Central South University, Changsha, China; ^3^ Xiangya School of Medicine, Central South University, Changsha, China; ^4^ Department of Pediatrics, The 3rd Xiangya Hospital, Central South University, Changsha, China

**Keywords:** COVID-19, delayed-onset PTSD, psychological health, college students, coping strategies

## Abstract

Coronavirus disease 2019 (COVID-19) has become a pandemic, and many Chinese college students both in China and abroad were house-quarantined. This study aimed to investigate the prevalence and symptoms of delayed-onset post-traumatic stress disorder (PTSD) and coping strategies among Chinese overseas and domestic college students during this pandemic. A questionnaire was opportunistically distributed to Chinese college students studying both domestically and abroad six months after the COVID-19 outbreak. The questionnaire consisted of IES-R, SCSQ, and SSRS. The average score of delayed-onset PTSD in our population was 21.411 (full mark, 88 points), which reflected a total high level of delayed-onset PTSD symptoms. Statistical differences were shown between students who have been back to universities during the pandemic or not in the hyperarousal dimension (*p* = 0.016). Three coping strategies were recognized to influence the respondent’s delayed-onset PTSD symptoms, and there was a significant correlation between social support and the coping strategies students chose. A moderate to high level of delayed-onset PTSD was observed among both Chinese overseas and domestic college students 6 months after the COVID-19 outbreak. The useful coping strategies and powerful social supports are significantly important to help them stay mentally healthy and alleviate delayed-onset PTSD during the COVID-19 pandemic.

## Introduction

The coronavirus disease 2019 (COVID-19) outbreak, which began in late 2019, has become a global pandemic. Severe acute respiratory syndrome–related coronavirus 2 (SARS-CoV-2) is a virus transmitted by inhalation or contact with infected droplets with an incubation period ranging from 2 to 14 days (Zhang et al., 2020a). As of September 31, 2020, there have been 33, 145, 948 confirmed cases worldwide (COVID-19 Dashboard by the Center for Systems Science and Engineering (CSSE) at Johns Hopkins University (JHU), 2020). The susceptibility and transmissibility of the virus had a huge impact on many activities around the world, including medical education. It was announced that over 1 billion students across the planet have been affected by school and college closures during this pandemic (Fatani, 2020).

The rapid evolution during the pandemic, including travel restrictions and the closure of educational institutions across the country, has influenced the students of all age-groups (Chaturvedi et al., 2021). Although the epidemic situation in China has been alleviated to some extent and many students have returned to school, the epidemic still affects the mental health status of teachers and students in universities (Hjiej and Fourtassi, 2020). Till now, most universities are still blocked down, disapproved of students going out of school. They need to update their body temperature every day and wear face masks every day when they have classes. We think it might have influences on student’s mental situation between Chinese students who have gone back to university at least once and those who never went back to university during the pandemic. In addition, many overseas students are facing the problem of being unable to return to school and continue their online courses (Smith, 2020). Meanwhile, some overseas students were also facing the situation that they cannot come back to China because of travel restrictions or high price tickets (Nicola et al., 2020). Such circumstances might have hatched the delayed-onset post-traumatic stress disorder among students.

Post-traumatic stress disorder (PTSD) refers to an abnormal mental reaction, responding to the severe traumas or disasters. Delayed-onset PTSD is designed to emphasize that “at least six months elapsed between the traumatic event and the onset of symptoms.” This is going to be a long-lasting effect. Given that the global COVID-19 pandemic has been lasting for nearly two years, the impact on people from the first outbreak in late 2019 till now would meet the definition of “delayed onset.” The death toll from COVID-19 continues to increase, with intermittent regional outbreaks. Such a situation constantly disturbs people’s normal work and life, causing people to continue to stimulate, a long-term traumatic stress reappearance state.

There are differences between China’s pandemic situation and other countrie’s, but we can still find something in common. In the COVID-19-PTSD scoring experiment of Italians, 29.5% of the population had PTSD symptoms. According to this study, the COVID-19 pandemic can be considered as “a traumatic event” (Forte et al., 2020). Liang et al. (2020) found that nearly 40.4% of the sampled youth were prone to have psychological problems, and 14.4% showed PTSD symptoms. Even in the home-quarantined college students, PTSD and depression prevalence were found to be 2.7 and 9.0%, respectively, followed by situations like sleep durations (Tang et al., 2020a). PTSD symptoms often come out 1–6 months after the triggering event. Delayed-onset PTSD, nonetheless, may be easily overlooked and diagnosed over 6 months after the traumatic event or termination of a long-term exposure (Utzon-Frank et al., 2014), and as college students are relatively unstable both physically and mentally, and they are facing some stressors, which are new for them, without enough family supports compared to high school, they are a group which might be easy to get PTSD symptoms or even get delayed-onset PTSD (Read et al., 2011; Ahmad et al., 2018). The study conducted by Fu et al. (2013) found that at one year after the Wenchuan earthquake in China, the PTSD rate in college students in the affected area was relatively higher.

Social support and anxiety levels were found to be negatively correlated during the COVID-19 epidemic (Cao et al., 2020). The previous research results of our research group also suggested that the main appeal of medical students and medical researchers to relieve high pressure is to resume normal scientific research and learning activities as soon as possible (Zhang et al., 2020b). In order to further analyze the situation and possible causes of PTSD after six months of the COVID-19 outbreak, we thus conducted a further questionnaire survey to judge the long-term impact of the epidemic on the student’s mental health. We designed this experiment in order to demonstrate the prevalence and symptoms of delayed-onset PTSD during the COVID-19 pandemic, providing relevant data and effective coping strategies for schools and health departments that may prevent delayed-onset PTSD among college students. It may help to provide a new perspective for schools to prevent students from developing delayed-onset PTSD and help students to better adjust themselves.

## Materials and Methods

### Study Participants

A questionnaire was distributed to both Chinese students who study at home and abroad to recruit an opportunity sample, the duplicated entries are not allowed, and each IP can answer only once. All respondents were asked to answer each question on their own. The targets of the questionnaire were identified as “Chinese students both study at home and abroad,” including four types: 1) overseas students who are still abroad (including visiting scholars), 2) overseas students who have returned to China (including visiting scholars), 3) Chinese students who studied in China (have been back to university once), and 4) Chinese students who studied in China (never been back to university during pandemic).

### Data Collection

Data were collected by a convenience sample method between July 31 and August 9, 2020. Questionnaires were distributed to college students (mainly medical major, as well as other majors), international students, and visiting scholars with Chinese nationalities through WeChat or QQ (the most commonly used mobile applications among Chinese), and they were asked to join anonymously to complete the questionnaire. At the end of the questionnaire, question No. 6 was repeated. If the respondents show different answer in these two questions, their questionnaires would have been excluded. A total of 344 survey copies were collected, and 319 valid questionnaires were recovered. All participants provided written informed consent. It is not required by our institution to obtain ethical approval for a survey with a non-clinical sample and anonymized data, but we did obtain approval for the study protocol from the Institutional Review Board (Ethics Committee) of the Third Xiangya Hospital of Central South University (20005-IRB).

### Questionnaire

#### Impact of Event Scale-Revised (IES-R)

The PTSD symptoms during the 6-month period following the COVID-19 outbreak were measured by IES-R (Beck et al., 2008). The IES-R has 22 items, each with a Likert scale rating from 0 to 4. The total score has a range of 0–88. The IES-R has been translated into and validated in Chinese (Chong et al., 2004). A score of 20 or more was interpreted here—as suggested by previous studies of populations affected by traumatic events (Hawryluck et al., 2004)—to indicate a high level of PTSD symptoms.

#### Simplified Coping Style Questionnaire

The coping strategies were measured by SCSQ (Sun et al., 2019). The SCSQ, based on the Ways of Coping Questionnaire (YN, 1998; Wang et al., 2020), is a 20-item instrument consisting of two subscales, positive coping (12 items) and negative coping (eight items). Each item of the SCSQ was ranked on a 4-point Likert scale ranging from 0 point to 3 points. The SCSQ had adequate content validity, internal consistency, and test–retest reliability in Chinese.15 In this study, Cronbach’s alpha of the positive coping and negative coping was 0.904 and 0.877, respectively.

#### Social Support Rating Scale

Social support was measured by SSRS (Xiao et al., 2020). The SSRS was developed by Xiao et al. with an acceptable validity and reliability (SY, 1994). The SSRS is a 10-item instrument consisting of three subscales, objective social support (three items about living conditions in the past year, problem-solving channels in emergency situations, and sources of psychological comfort in the event of stress or resistance), subjective social support (four items about relationship with colleagues, relationship with neighbors, number of friends who can offer assistance, and level of support from family members), and support utilization (three items about the way one talks when in trouble, the way one asks for help when in trouble, and participation in group activities). Higher scores indicate higher levels of social support. In the present study, Cronbach’s alpha of the three subscales ranged from 0.678 to 0.756.

### Demographic Characteristics and Self-Made Questions

Data such as gender, age, educational attainment, and school locations were collected. Questions No. 6 and 7 aimed at investigating what facts stress students the most. Question No. 19 was the same as the No. 6 to simply test the internal consistency of the questionnaire. Two open questions have been included to collect respondent’s individual ways of coping stress and what other support they need to release stress.

### Statistical Analysis

First, we described the data using numbers and percentages to demonstrate the demographic characteristics of respondents. Second, the means, standard deviations (SD), and ranges of the IES-R items have been used for continuous variables and proportions for categorical variables. We then conducted the *t*-test to contrast the difference between student’s PTSD in different situations. Third, the logistic regressions have been used to explore the association between student’s PTSD symptoms and the coping strategies they used. Fourth, we did correlation between the student’s social support and coping strategies to dig out the connection between them.

Questionnaire results were summarized from the imported Excel file and analyzed using SPSS version 18.0 software. A *p* value less than or equal to 0.05 was considered statistically significant.

## Results

### Basic Information

319 valid questionnaires from 344 respondents were received in our study, and 79.94% of the subjects were aged between 18 and 24 years. The age distribution is broadly consistent with the educational background, which suggests that the majority should be undergraduates or master candidates, the rest being PhD, postdocs, or visiting scholars. 85.90% of the respondents were domestic students, while 14.11% were overseas. Among the 45 overseas students, 91.11% were studying in North America and Europe. The specific information of the respondents is shown in Table 1.

**TABLE 1:** Demographic characteristics of respondents.

Characteristics		N	%
Age (years), *n* = 319	≥18 and <24	255	79.94
	≥24 and <35	60	18.81
	≥35 and <40	3	0.94
	≥40	1	0.31
Gender	Male	123	38.56
(*n* = 319)	Female	196	61.44
Education background	Undergraduates	190	59.56
	Master candidate	80	25.08
(*n* = 319)	PhD candidate	45	14.11
	Post-doctorate candidate	4	1.25
Situations during pandemic	Overseas students who are still abroad (including visiting scholars)	22	6.9
	Overseas students who have returned to China (including visiting scholars)	23	7.21
(*n* = 319)	Chinese students who studied in China (have backed to university once)	82	25.71
	Chinese students who studied in China (never backed to university during pandemic)	192	60.19
Location of overseas students	Asian	1	2.22
(*n* = 45)	The North American	24	53.33
	The European (including Russia)	17	37.78
	Oceania	3	6.67
	Others	0	0

### The Stressful Factors of the National and International Students

**FIGURE 1:**
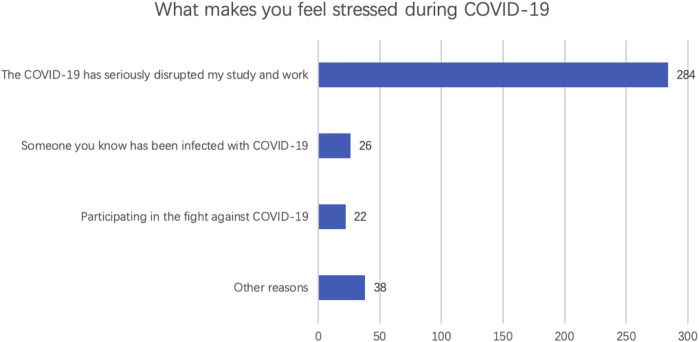
Stressful factors of the national and international students (*n* = 319).

Three main factors were considered by students to possibly stress them (Figure 1). The top one factor was “COVID-19 has seriously disrupted my study and work,” with 284 of 319 (89.03%) respondents regarding it stressful.

**FIGURE 2:**
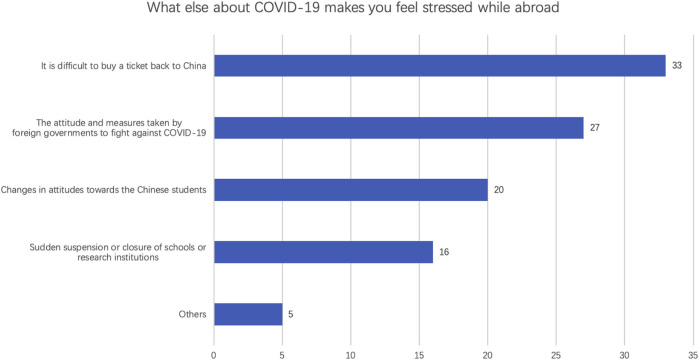
Special stressful factors of the international students (*n* = 45).

There are 45 respondents who studied international. 33 of 45 respondents reported “it is difficult to buy a ticket back to China” (Figure 2). 60% international students worried about the attitude and measures taken by foreign governments to fight against COVID-19, in which 60% students studied in North America. The following factor is the attitude’ change toward Chinese students while abroad (20 of 45). But only 35.56% respondents felt stressful from the sudden suspension or closure of schools or research institutions.

### The Persistence of Delayed-Onset PTSD Symptoms in Students

After 6 months of the outbreak, the average score of the population’s PTSD level was 21.411 (full mark, 88 points), which is more than 20, can be indicating a total high level of PTSD symptoms. 168 of 319 respondents scored more than 20 points. Moreover, 22 respondents scored more than 44 points, which can be identified as severe PTSD symptoms. In the avoidance part, the average score of students was 8.295 points (full mark, 32 points), which is relatively higher than that of other two dimensions. Item 1 in the intrusion dimension got the highest mean score (1.519 score), followed by 1.408 in item 5 (Table 2).

**TABLE 2:** IES-R items of the respondents (*n* = 319).

Items	Range	Mean	SD
Intrusion	0–32	8.016	5.607
1. Any reminder brought back feelings about it	0–4	1.589	0.950
2. I had trouble staying asleep	0–4	0.865	0.957
3. Other things kept making me think about it	0–4	1.113	0.948
6. I thought about it when I didn’t mean to	0–4	1.078	0.943
9. Pictures about it popped into my mind	0–4	0.972	0.953
14. I found myself acting or feeling like I was back at that time	0–4	0.821	0.863
16. I had waves of strong feelings about it	0–4	0.887	0.921
20. I had dreams about it	0–4	0.690	0.866
Avoidance	0–32	8.295	5.997
5. I avoided letting myself get upset when I thought about it or was reminded of it	0–4	1.408	1.112
7. I felt as if it hadn’t happened or wasn’t real	0–4	0.900	0.956
8. I stayed away from reminders about it	0–4	0.834	0.898
11. I tried not to think about it	0–4	0.956	0.983
12. I was aware that I still had a lot of feelings about it, but I didn’t deal with them	0–4	0.978	0.976
13. My feelings about it were kind of numb	0–4	1.480	1.129
17. I tried to remove it from my memory	0–4	0.856	0.920
22. I tried not to talk about it	0–4	0.884	0.988
Hyperarousal	0–24	5.100	4.327
4. I felt irritable and angry	0–4	1.041	0.922
10. I was jumpy and easily startled 15. I had trouble falling asleep	0–4	0.715	0.826
15.I had trouble concentrating	0–4	0.765	0.927
18.Reminders of it caused me to have physical reactions, such as sweating	0–4	1.085	1.004
19. trouble breathing, nausea, or a pounding heart	0–4	0.621	0.815
21. I felt watchful and on guard	0–4	0.875	1.005
Total	0–88	21.411	15.016

Analyzing and comparing Chinese students who have been back to school during the pandemic or not (Table 3), it showed that the students who had never been back to their universities during the pandemic got more serious PTSD symptoms (21.95 ± 14.79), and there were statistical differences between these two groups in the hyperarousal dimension (*p* = 0.016). We did not report the IES-R ANOVA analysis other than these two groups in which there was no significant difference.

**TABLE 3:** The comparisons of PTSD between Chinese students who has been back to school during pandemic or not.

IES-R	Chinese students who studied in China (means ± SD)		t	p
	Back to university once (*n* = 82)	Never back to university during pandemic (*n* = 192)		
Avoidance	7.06 ± 5.81	8.49 ± 6.01	−1.827	0.069
Intrusion	6.91 ± 5.01	8.15 ± 5.56	−1.735	0.084
Hyperarousal	3.99 ± 3.90	5.30 ± 4.20	−2.42	0.016*
Total scores	17.96 ± 13.81	21.95 ± 14.79	−2.082	0.038*

*p < 0.05 **p < 0.01

### The Coping Strategies for the COVID-19 Pandemic

The original SCSQ has divided all the coping strategies to positive and negative sections. The items are listed in Table 4. The median of item “I tried to get away from it by eating, drinking, smoking, using drugs or medicine, *etc*.” is 0. 56.1% respondents reported never use this way to cope with stress.

**TABLE 4:** The coping strategies of the respondents (*n* = 319).

Coping strategies	Range	Means	SD	Median
Positive	0–3	1.985	0.652	2
I tried to make myself feel better by working, studying, etc.	0–3	1.912	0.843	2
I asked advice from a relative, friend or classmate	0–3	1.774	0.860	2
I tried to look on the bright side of things	0–3	2.128	0.767	2
I changed something about myself	0–3	2.000	0.834	2
I didn’t take it too seriously	0–3	2.044	0.820	2
I made a plan of action and followed it	0–3	2.069	0.774	2
I found new faiths to solve the problem	0–3	2.007	0.794	2
I confided my troubles to my family, friends or colleagues	0–3	1.920	0.865	2
I changed or grew as a person in a good way	0–3	1.934	0.748	2
I drew on others experiences in the similar situation	0–3	1.912	0.751	2
I tried to make myself feel better by engaging in hobbies, leisure activities, and recreation	0–3	1.916	0.875	2
I tried to keep my feelings (e.g., sadness and anger) to myself	0–3	1.865	0.864	2
Negative	0–3	1.369	0.622	1
I tried to get away from it for a while by resting or taking vacation	0–3	1.883	0.835	2
I tried to get away from it by eating, drinking, smoking, using drugs or medicine, etc.	0–3	0.639	0.900	0
I was waiting for time to change the situation	0–3	1.551	0.957	2
I refuse to think too much about it	0–3	0.821	0.934	1
I relied on others to solve the problem	0–3	1.077	0.811	1
I accepted this situation because there is nothing I can do to change it	0–3	1.679	0.881	2
I had fantasies or wishes about how things might turn out	0–3	1.084	0.920	1
I went along with fate, sometimes I just have bad luck	0–3	1.766	0.867	2

The whole coping strategie’s list can explain the 24.4% students have the PTSD trend. We identified three coping strategies (Table 5) which influenced the respondent’s PTSD symptoms. In the list of positive items, it showed that the more one made a plan and followed it in one’s daily life, the less PTSD one would have (t < 0 and *p* = 0.033). The item in the negative coping strategies, if you kept refusing to think anything about COVID-19, the PTSD situation might get worse (t > 0 and *p* = 0.001). However, the item “I confided my troubles to my family, friends, or colleagues,” which in the positive coping strategies list, also had positive correlation to the PTSD level (t > 0 and *p* = 0.007 < 0.005). This result might be related to the special “stay home” policy implemented in China.

**TABLE 5:** The regressions between coping strategies and respondent’s PTSD symptoms.

	Unstandardized coefficient		Standardized coefficient	t	p	VIF	*R* ^2^	Adjusted *R* ^2^	F
	B	SE	ß						
Constant	14.125	3.091	–	4.569	0.000**	–	0.242	0.182	F (20,253) = 4.029, P< 0.05
I tried to make myself feel better by working, studying, etc.	0.391	1.307	0.023	0.299	0.765	1.9			
I confided my troubles to my family, friends or colleagues	3.557	1.307	0.21	2.722	0.007**	1.978			
I tried to look on the bright side of things	0.727	1.609	0.038	0.452	0.652	2.383			
I changed something about myself	−2.313	1.584	−0.132	−1.461	0.145	2.734			
I didn’t take it too seriously	−1.703	1.387	−0.096	−1.228	0.221	2.025			
I made a plan of action and followed it	−3.369	1.572	−0.179	−2.143	0.033*	2.321			
I found new faiths to solve the problem	0.864	1.774	0.047	0.487	0.626	3.103			
I confided my troubles to my family, friends or colleagues	−0.415	1.354	−0.025	−0.306	0.76	2.147			
I changed or grew as a person in a good way	1.474	1.638	0.076	0.899	0.369	2.353			
I drew on others experiences in the similar situation	−0.764	1.651	−0.039	−0.463	0.644	2.407			
I tried to make myself feel better by engaging in hobbies, leisure activities, and recreation	−0.943	1.405	−0.057	−0.671	0.503	2.369			
I tried to keep my feelings (e.g., sadness and anger) to myself	0.37	1.315	0.022	0.282	0.778	2.023			
I tried to get away from it for a while by resting or taking vacation	2.312	1.258	0.132	1.838	0.067	1.726			
I tried to get away from it by eating, drinking, smoking, using drugs or medicine, etc.	0.948	1.118	0.058	0.848	0.397	1.586			
I was waiting for time to change the situation	−0.67	1.095	−0.044	−0.612	0.541	1.718			
I refuse to think about it	4.136	1.177	0.265	3.514	0.001**	1.893			
I relied on others to solve the problem	−0.587	1.295	−0.033	−0.453	0.651	1.724			
I accepted this situation because there is nothing I can do to change it	0.12	1.163	0.007	0.104	0.918	1.643			
I had fantasies or wishes about how things might turn out	1.71	1.143	0.108	1.495	0.136	1.733			
I went along with fate, sometimes I just have bad luck	1.785	1.209	0.106	1.476	0.141	1.719			

*p < 0.05, **p < 0.01.

### The Relationship Between Student’s Tendency to Take Positive Measures and Social Support

According to Table 6, there is a significant correlation between social support and coping strategy students choose. In particular, the three dimensions of objective social support, subjective social support, and support utilization were positively correlated with the level of positive measures students chose to take (*p* < 0.05). There were also differences in social support between domestic students and overseas students (Table 7). The total scores of domestic students (27.07 ± 4.13) were higher than those of foreign students (25.13 ± 4.87), and the scores of objective social support, subjective social support, and support utilization of domestic students are, respectively, higher than the latter.

**TABLE 6:** Correlations between social supports and coping strategies of the participants (*n* = 319).

	Mean	SD	Positive coping	Negative coping
Subjective social support	8.276	1.717	0.169**	0.113*
Objective social support	9.715	2.268	0.242**	0.023
Support utilization	8.803	1.536	0.282**	0.181**
Total scores	26.793	4.288	0.302**	0.122*

*p < 0.05 **p < 0.01

**TABLE 7:** Comparison of social supports between oversea students and students who study in China.

	Students (Mean ± SD)		t	p
	Overseas students (*n* = 45)	Domestic students (*n* = 274)		
Objective social support	7.36 ± 1.80	8.43 ± 1.66	−3.968	0.000**
Subjective social support	9.31 ± 2.26	9.78 ± 2.27	−1.29	0.198
Support utilization	8.47 ± 1.83	8.86 ± 1.48	−1.586	0.114
Total scores	25.13 ± 4.87	27.07 ± 4.13	−2.832	0.005**

*p < 0.05 **p < 0.01

## Discussion

The average score of delayed-onset PTSD in our respondents was high, and in complex stressors for students, most respondents reported “COVID-19 has seriously disrupted my study and work” was stressful. The students who have been back to universities during the pandemic got less score in the hyperarousal dimension of IES-R. After testing the regressions between coping strategies and respondent’s PTSD symptoms, our study finds that the best coping strategy is to do planning and manage their life during the pandemic, and there was a significant correlation between social support and the coping strategies students chose.

Delayed-onset PTSD is a real and potentially sizable problem that could easily be overlooked if one only focused on the first one to three months after the outbreak (Andrews et al., 2007). Andrews et al. (2009) found that the delayed onsets were more likely to report the presence of severe stressor before onset than the immediate-onset PTSD. At the beginning of the COVID-19 outbreak, college students were facing complex stressors according to our investigation, including but not limited to the new online study systems, the delay of their research, and home quarantine policy. Thus, the PTSD symptoms caused by COVID-19 cannot be recognized but attributed to other pressure. As time goes by, the students did not recover from the anxiety but reveal more relevant PTSD symptoms (Chi et al., 2020). Our study shows high to moderate levels of PTSD among college students 6 months after the COVID-19 outbreak, while one month after the outbreak, the PTSD prevalence was found to be low according to Tang et al. (2020b). On the one hand, universities in China generally prohibited students from returning universities, which prolong the student’s home time. Our research showed that, compared to those students who have been back to school, those who have never been back had severe PTSD. Henderson and Redshaw (2017) described the negative effects of long-term staying at home for women, including confusion, anger, resentment, and feeling neglected, unsupported, and anxious. We thought the same mental situation was happening on students who have never been back to school during the pandemic 6 months after the outbreak. On the other hand, the disruption to student’s study caused by COVID-19 is the top one reason which made all of our candidates feel stressful and anxious. Some universities in China adopt the form of online course teaching, but online education still has some limitation. For example, the internship and the laboratory work for some students majored in medicine or biology have been limited or canceled. Especially for students in the graduation grade, they might need to face the pause or change of their research progress for graduation, which makes them stress a lot (Araz Altay et al., 2020).

For overseas Chinese students, there are some special stressful reasons that occurred, including the shortage of tickets back to China, the different attitude and measures taken by foreign governments, and the change of local’s attitude toward them. These long-term persistent stress factors contribute to the overseas student’s PTSD as they can hardly leave the country where COVID-19 broke out seriously in a timely manner. How to live through a long period of isolation and how to protect themselves from the virus have become a survival problem for many overseas students.

To help investigate the useful coping strategies for students to alleviate the PTSD symptoms, we asked candidates to evaluate their coping strategies by SCSQ. The action “I made a plan of action and followed it” is also positive to decrease the PTSD. It gave students one more reason to manage their life and do planning. As we all know, the social life and frequent contact with family members are necessary for college students to release pressure. However, in this study, we find that those students who confided their troubles to the family, friends, or colleagues more responded more PTSD symptoms. First, the stress caused by the pandemic is not a personal trouble, it related to everyone. Yuan et al. found that there is a general increase in people’ anxiety. When students confided it to the people around, they might get some new stressors instead of being well comfort (Yuan et al., 2020). Second, people have been encouraged to be indoors to prevent virus during the pandemic, which largely increase the family time. For some families, it might tense the family and make everyone anxious. From the college’s point of view, we suggest the emphasis on teaching students to balance their social network. Most of the time, confiding the troubles to the family, friends, or colleagues is useful to keep mental health, but students also need to know how to cheer up themselves and people around when general anxiety happened. Karyotaki et al. (2019) mentioned that college students experience a variety of stressors (e.g., exams, living away from family, and financial hardships), which make them prone to mental disorders. As a result, the strategies mentioned above can not only be useful in decreasing PTSD caused by the COVID-19 outbreak but also be consistently used in students’ daily life.

We also find that the objective social support is negatively correlated with the PTSD of students. Objective social support focus on the direct material assistance and the presence and participation of social networks and group relationships. Compared to domestic students, overseas Chinese students usually get less objective social support as they are far away from homeland and live by themselves. Back in 2008, Xu et al. demonstrated that when students face the catastrophic earthquake emergency, effective coping strategies can help to protect students’ mental health (Xu and He, 2012). In a cross-sectional and longitudinal study of veterans, Matthew et al. found that social support moderated changes in PTSD symptoms, underscoring the important link between social support and symptom improvement during PTSD treatment (Price et al., 2018). On this basis, the impact of social support on the individual becomes particularly important. We suggest the international students to strengthen their subjective social support and support utilization, like creating more chances to feel been respected and supported like doing more volunteer work, contacting their close friends or families, and finding their interests. These are all consistent with our experimental results.

The study has several limitations. First, the opportunity sampling method has been used to select participants from a target group. It is a popular technique, especially among researchers who may have limited resource (like the quarantine strategy in COVID-19 provides us less chance to select a truly random sample). The downside of this method is that the researches may end up with biased results. Second, the COVID-19 outbreak burst not at the same time worldwide, which might cause the delay of the delayed-onset PTSD in overseas students. It is better to evaluate overseas student’s delayed-onset PTSD again after 6 months, and the tendency of students to take negative behaviors is also correlated with the degree of social support to some extent, which may result in the unclear definition of negative coping measures in the items of the scale. Third, the limitation of network-based questionnaire survey is that most questionnaires are spread spontaneously in the circle of friends. There is a certain possibility that the psychological changes of people who share the circle of friends may influence each other, leading to the convergence of psychological states presented in the survey. Although we have made the limitation of questionnaire fillers, its coverage is still limited compared with the paper questionnaire we made in the past. In the next experiment, if we try to further explore the correlation between negative behaviors and social measures, it is better to redefine and re-list negative coping measures in a more detailed and clear way.

## Conclusion

College students show high to moderate levels of delayed-onset PTSD for 6 months after the COVID-19 outbreak. The long-term disruption of the study process is a main reason to student’s delayed-onset PTSD. Our study finds that the best coping strategy is to plan and manage their life during the pandemic. The results show that social support is negatively correlated with the PTSD of students. We suggest colleges emphasize the social support education since the first year of students, and for overseas students, they now might still face an uncontrolled outbreak in other countries and some special stressors, like the lack of back-home ticket and the local’s changing attitude toward Chinese. The useful coping strategies and powerful social supports are significantly important to help them stay mentally healthy and alleviate delayed-onset PTSD caused by the COVID-19 outbreak.

## Data Availability

The raw data supporting the conclusions of this article will be made available by the authors, without undue reservation.

## Abbreviations

Delayed-onset PTSD, delayed-onset post-traumatic stress disorder; COVID-19, coronavirus disease 2019; IES-R, impact of event scale-revised; SCSQ, simplified coping style questionnaire; SSRS, social support rating scale.
